# Isolation and comparison of neural stem cells from the adult rat brain and spinal cord canonical neurogenic niches

**DOI:** 10.1016/j.xpro.2022.101426

**Published:** 2022-06-07

**Authors:** Lars Erik Schiro, Ulrich Stefan Bauer, Axel Sandvig, Ioanna Sandvig

**Affiliations:** 1 Department of Neuromedicine and Movement Science, Faculty of Medicine and Health Sciences, Norwegian University of Science and Technology (NTNU), 7030 Trondheim, Norway; 2 Department of Clinical Neuroscience, Division of Neuro, Head, and Neck, Umeå University Hospital, 907 37 Umeå, Sweden; 3 Department of Community and Rehabilitation, Division of Neuro, Head and Neck, Umeå University Hospital, 907 37 Umeå, Sweden

**Keywords:** Cell Biology, Cell culture, Cell isolation, Microscopy, Model Organisms, Neuroscience, Stem Cells

## Abstract

Here, we present a unified protocol for the extraction, culture, and basic characterization of rat neural stem cells (NSCs) from all three canonical neurogenic niches in the brain and spinal cord. We describe tissue dissection and dissociation, cell culture, followed by EdU labeling and characterization of NSCs. By yielding considerable numbers of viable cells per animal, this protocol enables the establishment of substantial, long-term cell banks, thus offering cost and labor efficiency while significantly reducing the numbers of animals used.

## Highlights

• Extraction, culture, and characterization of adult neurogenic niche neural stem cells
• Comparison of neural stem cells from the different neurogenic niches
• High yield of viable neural stem cells obtainable per animal

## Abstract (editor highlights)

Publisher’s note: Undertaking any experimental protocol requires adherence to local institutional guidelines for laboratory safety and ethics.

## Graphical abstract

**Figure:**
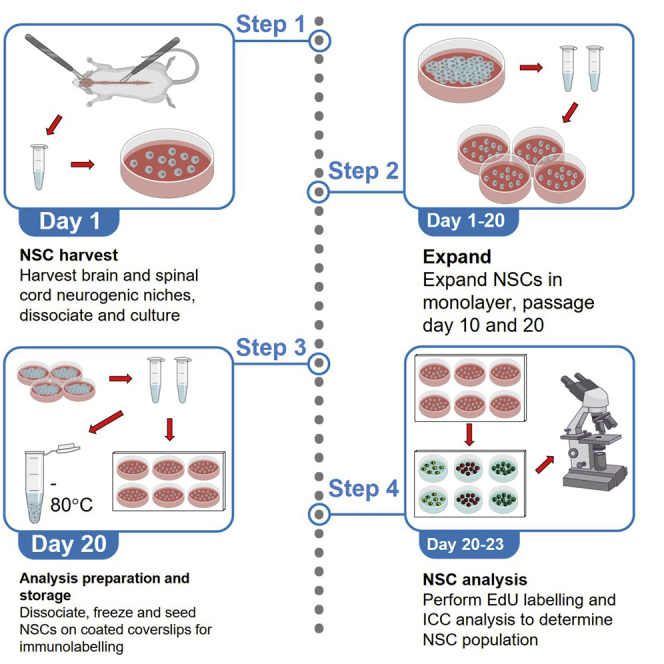

## Before you begin

**Timing: 1–2 h**

**CRITICAL:** This protocol is optimized for NSC harvest from 10-week-old female Sprague Dawley rats for each of the three neurogenic niches; the subventricular zone (SVZ) and subgranular zone (SGZ) in the brain, and the central canal (CC) of the spinal cord. The principles described in this protocol can be used for other rat strains, gender, and ages, however, the yields of viable NSCs may somewhat vary.

This protocol is optimized for the extraction of a single neurogenic niche per animal. However, we have also included guidance about how to extract both brain neurogenic niches (SVZ and SGZ) from the same animal. We **do not** however recommend extracting all three neurogenic niches from the same animal or extracting the CC in addition to either of the brain neurogenic niches (SVZ or SGZ) due to the impact of time-consuming procedures, i.e., euthanasia, dissection, and tissue extraction, on cell viability.

1. Aliquot sterile 10% Bovine Serum Albumin (BSA) solution into 500 μL aliquots for use in step 2. Freeze unused aliquots at −20°C.

***Note:*** Depending on the manufacturer, frozen BSA may be stable up to 24 months, see manufacturer’s instructions/certificate of analysis (CoA) of the particular batch.

2. Dilute 10% BSA in sterile DPBS −/− to a concentration on 0.1% BSA.
  a. Reconstitute Epidermal Growth Factor (EGF) and Fibroblast Growth Factor-basic (bFGF) using this 0.1% BSA solution, both to a concentration of 10 μg/mL, up to a total of 10 mL of each solution.
  b. Aliquot and store at −20°C.

***Note:*** Using a 0.1% BSA solution is recommended at this concentration. Check the recommended reconstitution buffer and carrier protein for dilution to lower concentrations on the manufacturer’s instructions/CoA for the particular batch of growth factor being used. Depending on the batch, frozen aliquots will typically be stable for 6–12 months.

**CRITICAL:** As thawed aliquots should be kept no longer than 7 days at 4°C, to ensure sufficient growth factor activity, the size of the aliquots should be adjusted based on the number of dissections and thereby wells of media that will need to be supplemented. For example, for the dissection and processing of one neurogenic niche, 50 μL aliquots would be a good volume.

3. Dissolve CHIR 99021 (Critical component: https://www.medchemexpress.com/CHIR-99021.html?src=google-product&gclid=CjwKCAjwmeiIBhA6EiwA-uaeFT5P4u01KjY5nIsLGeiDWiDpqpCIzIfMZcffoyhRuKuvm3SpdDxd-hoCP24QAvD_BwE) in DMSO to a final concentration of 10 mM. Aliquot and store at −80°C.

**CRITICAL:** CHIR 99021 can be stored at −80°C for up to 6 months, storage at −20°C reduces this to 1 month. Aliquots should be made for use within 7 days of thawing if stored at 4°C. Aliquot size should be adjusted accordingly, for example for 1 dissection 15 μL aliquots would be a good volume.

### Institutional permission

The animals were housed at the Comparative Medicine core facility, NTNU, with ad libitum access to food and water throughout the study. All animal experiments were approved by the Norwegian National Food and Health Safety Department (study ID: 18066 & 24506).

### Dissection preparation

**Timing: 20 min**

4. Fill a styrofoam box with crushed ice, approximately 2–3 cm above the top and flatten it. This will serve as your dissection platform.
5. Fill 1 × 15 mL tube with 10 mL Neurobasal medium 1× for each niche to be extracted, label “transport” + name of niche, and place in ice in the corner of the box to keep chilled (90% of vial covered by ice).
6. Fill 1 × 15 mL tube with 12–14 mL saline and place together with transport media tube to chill.
7. Disinfect surgical equipment by submerging it in disinfectant while preparing everything else, for a minimum of 10 min.

**CRITICAL:** Autoclave all reusable surgical instruments after every dissection session.

**Figure 1:**
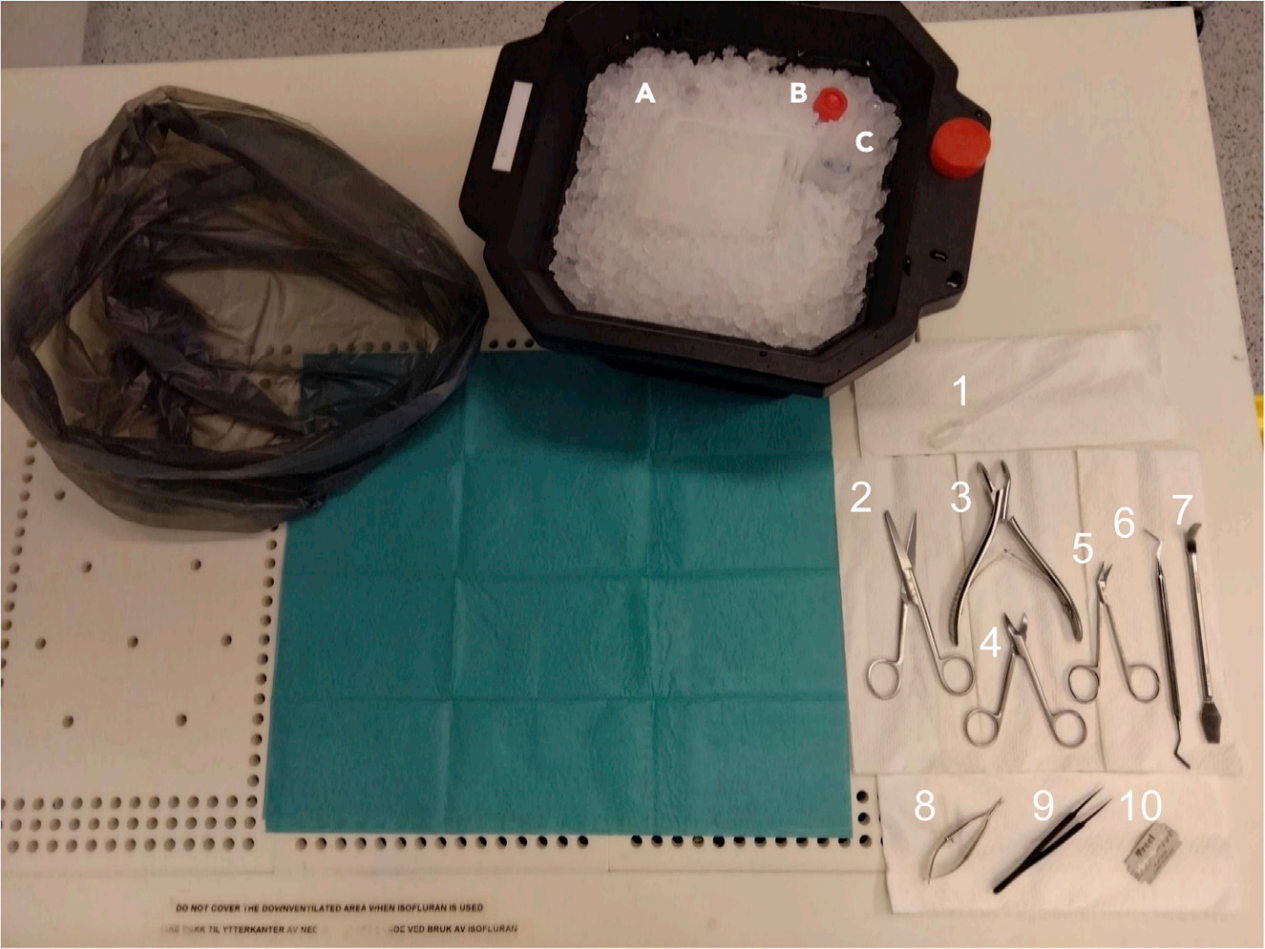
Suggested setup for dissection table Suggested setup for ease of access to all equipment and reagents required for efficient dissections. (A–C) (A) Wheaton Coplin staining jar lid with Bench Safe absorbent spillage protection soaked in chilled saline solution, (B) 15 mL conical tube with transport media, (C) 50 mL conical tube with sterile saline. 1) 1 mL disposable graduate transfer pipette, 2) Sharp/Blunt surgical scissors, 3) Rongeurs, 4) Bone scissors (curved), 5) Dissecting scissors, 6) Heidemann spatula, 7) Hippocampal dissection tool, 8) Vannas spring scissors, 9) Dumont #5 forceps, 10) Single use double-sided razor blade. Additional: Waste bag, Sterile OP-Towel, Ice box, crushed ice, and paper towels (optional).

8. Cut up filter paper into 5–7 cm^2^ pieces.

9. Prepare table for euthanasia and dissection as illustrated in Figure 1.
  **CRITICAL:** Ensure proper training has been completed and that the user fully understands the equipment before using gas anesthetics!
  a. Set up the guillotine, if possible, at a separate table/some distance away from the dissection space to avoid cross-contamination.
  b. Wipe off surgical equipment and place it at the table, taking particular care to wipe the disinfectant off the glass plate and razor blade.
  c. Place glass plate onto the ice platform on the styrofoam box, place a piece of filter paper on top of it and use a graduated transfer pipette to soak it in ice cold saline just before anesthetizing the animal.
    ***Note:*** Using the filter paper on top of the glass plate will hold tissue in place while dissecting. Do not use too much force when cutting as this can cut off fibers from the filter paper that can end up in the culture.
    **CRITICAL:** Remove filter paper before dicing the tissue with the razor blade.

### Preparation: Tissue dissociation (1 animal)

**Timing: 30–45 min**

**CRITICAL:** For media volumes < 10 mL, make 0.5 mL extra media. For media volumes > 10 mL, prepare another 1 mL of media for every 10 mL of base media.

Prepare 1 additional set of all reagents and materials if two niches (SGZ/SVZ) are to be extracted from the same animal.

10. Prepare NSC base media. Base media can be stored at 4°C for up to 7 days.
11. Prepare Resuspension and Expansion Media (Table 1), and supplement according to neurogenic niche of origin (Tables 2, 3, and 4). Allow to warm to 37°C in a water bath before starting the dissection.

**Table 1:** Expansion base media

Reagent	Final concentration	Amount
Neurobasal 1× Base medium	N/A	47.495 mL
B27 Without Vitamin A	1×	1 mL
N2	1×	0.5 mL
L-Glutamine	1×	0.5 mL
Penicillin-Streptomycin	1×	0.5 mL
Heparin	2.5 μg/mL	0.5 μL
**Total**		**50 mL**

Can be stored at 4°C for up to 1 week.

**Table 2:** Expansion media - Central Canal (CC)

Reagent (stock conc)	Final concentration	Amount
Expansion base media	N/A	3 mL/well
EGF (10 μg/mL)	40 ng/mL	12 μL/well
bFGF (10 μg/mL)	40 ng/mL	12 μL/well
**Total**		**≈3 mL/well**

Use immediately, do not store for any duration except warming as this will impact growth factor stability.

**Table 3:** Expansion media - Subventricular zone (SVZ)

Reagent (stock conc)	Final concentration	Amount
Expansion base media	N/A	3 mL/well
EGF (10 μg/mL)	40 ng/mL	12 μL/well
bFGF (10 μg/mL)	40 ng/mL	12 μL/well
CHIR 99021 (10 mM)	10 μM	3 μL/well
**Total**		**≈3 mL/well**

Use immediately, do not store for any duration except warming as this will impact growth factor stability.

**Table 4:** Expansion media - Subgranular zone (SGZ)

Reagent (stock conc)	Final concentration	Amount
Expansion base media	N/A	3 mL/well
EGF (10 μg/mL)	40 ng/mL	12 μL/well
bFGF (10 μg/mL)	40 ng/mL	12 μL/well
CHIR 99021 (10 mM)	10 μM	3 μL/well
**Total**		**≈3 mL/well**

Use immediately, do not store for any duration except warming as this will impact growth factor stability.

***Note:*** Prepare 1 mL Resuspension Media and 4 mL Expansion Media together per well (6-well plate) per dissection (this includes sufficient extra media as 3.5 mL is needed per well). Example: For 2 wells containing 1 million cells total, 10 mL total of Expansion and Resuspension Media should be prepared, and then aliquoted as indicated.

12. Pre-warm 12.5 mL of unsupplemented Neurobasal 1× medium as washing media, and heat to 37°C in a water bath before starting the dissection.
13. Disinfect a small Styrofoam box with 70% ethanol inside and out. Fill the disinfected box with crushed ice and spray down the ice with 70% ethanol before placing the box in the laminar flow cabinet.
14. Prepare a 15 mL tube with 11 mL HBSS.

***Note:*** Keep the HBSS−/− in either the ice box in the laminar flow cabinet or at 4°C and transfer to the ice box immediately before starting the dissociation.

15. Warm accutase for CC and SVZ and trypsin for SGZ dissociation to 37°C in a water bath immediately before use.

**CRITICAL:** Do no use trypsin for CC-derived cells as the cells do not tolerate it and will yield unviable cultures. Trypsin can also be used for SVZ-derived cells. The use of trypsin will impact the number of cells obtained, and may yield type-C-like cells. Accutase can be used for SGZ but will yield lower number of cells and reduce rate of proliferation in comparison to trypsin (Table 5 (old table 7)).

16. Set aside a sealed 0.5 mL microcentrifuge tube with 10 μL of 0.4% Trypan Blue.
17. Have all serological pipettes, an additional 15 mL tube and a cell counting chamber slide ready inside the laminar flow cabinet before starting.

### Preparation: Media change

**Timing: 30 min**

18. Prepare base media (Table 1) on the day of the 1^st^ media change, make new as needed throughout the expansion period.
19. Transfer 3 mL of base media per well to a 15 mL tube.
  a. Supplement base media with the specific growth factors required for each neurogenic niche (Tables 2, 3, and 4).
  b. Do this fresh before every media change for optimal stability of growth factors.

***Note:*** Use small aliquots, preferably to only cover 1–2 media changes (store thawed aliquots at 4°C; do not refreeze) so the rest can be stored frozen at -20°C for improved growth factor stability. CHIR 99021 aliquots should be stored at -80°C. If cells do not proliferate/proliferate at a slower pace or start spontaneously differentiating use aliquots of growth factors from a fresher batch as the contents may have degraded. Addition of 0.1% filtered BSA in the reconstitution solvent recommended by the manufacturer can help increase growth factor stability in storage and doesn’t interfere with this protocol.

20. Pre-warm media to 37°C in a water bath, prepare laminar flow cabinet and image cells during this time if tracking culture development is of interest.

### Preparation: Passaging

**Timing: 45 min**

21. Prepare NSC base media.
22. Prepare Resuspension and Expansion Media for the niche being passaged (Tables 2, 3, and 4). Supplement according to concentrations given for each niche in Table 6, allow to warm to 37°C in a water bath before starting the dissection.

**Table 5:** Expected yield from neurogenic niche harvest – live cell %

Neurogenic niche	Expected survival range (%)	Number of dissections
Central canal (accutase)	20–30	18
Subventricular zone (accutase)	60–80	17
Subventricular zone (trypsin)	5–50	2
Subgranular zone (accutase)	60–80	18
Subgranular zone (trypsin)	40–70	6

Optimal live cell % for harvest from each neurogenic niche, using either accutase or trypsin (as indicated). Higher or lower percentages may impact post-expansion NSC yields.

**Table 6:** Recommended cell seeding densities

Seeding target	Recommended seeding number
Full reseeding for expansion, 6-well plate	Total number of cells ÷ number of wells
Seeding for expansion, 6-well plate	5 × 10^5^/well
Seeding for differentiation, 6-well plate	5 × 10^5^/well
Seeding coverslips, 12-well plate	2.5 × 10^5^/well
Seeding coverslips, 24-well plate	1 × 10^5^/well
Seeding Microfluidics	2 × 10^4^/well
Cryogenic storage, 1 mL cryovial	1–3 × 10^6^/vial

**Calculation:** Find the average of total number of live cells ÷ 1000 for number of live cells/μL of cell resuspension solution. Number of cells to seed per well ÷ number of live cells/μL gives how many μL of cell solution contain the number of live cells to be seeded.

***Note:*** Prepare 1 mL Resuspension Media, and 3.5 mL Expansion Media per well of a 6-well plate per passage (this includes sufficient buffer as 3 mL is needed per well).

23. Pre-warm 12.5 mL of unsupplemented Neurobasal 1× medium as washing media, and heat to 37°C in a water bath before starting the passage.
24. Warm dissociation agent to 37°C in a water bath immediately before the passage.
25. Set aside a sealed 0.5 mL microcentrifuge tube with 10 μL of 0.4% Trypan Blue.
26. Have all serological pipettes, an additional 15 mL tube and a cell counting chamber slide ready inside the laminar flow cabinet before starting.

### Preparation: Cell seeding

**Timing: 3 h**

27. Prepare vessel of choice for culturing. The vessel used for this protocol is 13 mm glass coverslips in 24-well culture plates.
28. Place 1 coverslip in each well, submerge the coverslips by adding 300 μL Poly-L-Ornithine solution to each well, and incubate for 1 h (5% CO_2_, 37°C).
29. Wash coverslips 3 times for 5 min for each wash with sterile DH_2_O.
30. Prepare Laminin solution (Table 7) during step 29.

**Table 7:** Laminin solution

Reagent (stock conc)	Final concentration	Amount
L-15 Media	N/A	0.959 mL
Sodium Bicarbonate (0.9 M)	22.5 mM	25 μL
Natural Mouse Laminin (1 mg/mL)	16.5 μg/mL	16 μL
**Total**		**1 mL**

Use immediately, can be stored at 4°C for maximum 24 h. Be aware of batch variability when preparing Laminin stock solution.

**CRITICAL:** Prepare solution cold (Laminin will start to form a gel when heated, so avoid placing fingers on the bottom of the vial containing the solution) and store at 4°C for up to 24 h before use.

31. Submerge coverslips in Laminin solution and incubate for 1 h (5% CO_2_, 37°C).
32. Remove and replace Laminin solution with fresh and warm (37°C) appropriate cell culture media and place in incubator before starting passage.

### Preparation: EdU labeling

**Timing: 1 h**

33. 1 day before starting EdU labeling: Prepare EdU kit solutions in accordance with protocol provided by supplier: (https://www.thermofisher.com/se/en/home/references/protocols/cell-and-tissue-analysis/protocols/click-it-edu-imaging-protocol.html).

**CRITICAL:** Ensure proper protective gear is worn when handling EdU as it interacts with DNA synthesis.

### Preparation: Immunostaining

**Timing: 1.5 h**

34. Ensure that all reagents and antibodies for immunocytochemistry are available.
35. Image cells for references and prepare Glyoxal fixative (Table 8) to final concentration (3%) just before fixation.

**Table 8:** Glyoxal fixative

Reagent	Final concentration	Amount
DH_2_O	N/A	0.708 mL
Ethanol Absolute	N/A	0.197 mL
Glyoxal 40%	3%	0.078 mL
Acetic Acid	0.3%	7.8 μL
**Total**		**1 mL**

Use immediately, components can be stored at RT (20°C–23°C) for at least up to 6 months.
36. If staining is to be done directly following fixation, take out aliquots of goat serum to thaw at room temperature once cell fixation has been started. Also use this time to prepare Triton-X 100 solution (0.5% diluted in 1× PBS).

Set up workstation with base solution needed (recommended: 50 mL tubes for 1× PBS, DH_2_O and waste), pipettes and pipette tips.

## Key resources table

**Table:** 

REAGENT or RESOURCE	SOURCE	IDENTIFIER
**Antibodies**		

A2B5 Anti-Mouse (1:1000)	Abcam	ab53521
Beta-3-Tubulin Anti-Mouse (1:1000)	Abcam	ab78078
Dix2 Anti-Rabbit (1:500)	Abcam	ab272902
GFAP Anti-Rabbit (1:1000)	Abcam	ab7260
GFAP Anti-Chicken (1:1000)	Abcam	ab4674
Ki67 Anti-Rabbit (1:500)	Abcam	ab15580
Map2 Anti-Chicken (1:5000)	Abcam	ab5392
Nestin Anti-Mouse (1:250)	Abcam	ab6142
SOX2 Anti-Rabbit (1:100)	Abcam	ab92494
Alexa Fluor Goat Anti-Rabbit: 488 (1:500)	Abcam	ab150077
Alexa Fluor Goat Anti-Mouse: 568 (1:500)	Abcam	ab175473
Alexa Fluor Goat Anti-Chicken: 647 (1:500)	Abcam	ab150171
Hoechst 33342 (1:2000)	Invitrogen	C10337 G

**Chemicals, peptides, and recombinant proteins**		

Accutase	Gibco	A1110501
Acetic Acid	Merck	Cat# 64-19-7
B27 Without Vitamin A (50×)	Gibco	Cat# 12587010
B27 Plus (50×)	Gibco	A3582801
BDNF	PeproTech	Cat# 450-02
bFGF	Gibco	PHG0026
CHIR 99021	MedChem Express	HY-10182
Click-IT EdU Cell Proliferation kit	Thermo Fisher Scientific	C10337
Crushed ice (H_2_O, approx. size: 0.2–0.5 cm^3^)	N/A	N/A
DH_2_O	N/A	N/A
Dimethyl Sulfoxide (DMSO)	Sigma-Aldrich	D8418
EGF	Sino Biological	Cat# 10605-HNAE
Ethanol Absolute	Kemetyl	Cat# 200-578-6
Fetal Bovine Serum (FBS)	Sigma-Aldrich	F9665
Glyoxal 40% Stock	Sigma-Aldrich	Cat# 128465
HBSS −/−	Gibco	Cat# 14170-112
Heparin	Sigma-Aldrich	H3149
Isoflurane	Baxter	ESDG9623C
L-15 cell medium	Sigma-Aldrich	L5520
L-Glutamine (100×)	Gibco	Cat# 25030081
N2 (100×)	Gibco	Cat# 17502047
Nail kind nail polish topcoat	Alba beauty Nordics	Cat# 2820
Natural Mouse Laminin	Gibco	Cat# 23017-015
Neurobasal 1× Base medium	Gibco	Cat# 21103049
Normal Goat Serum	Abcam	ab7481
Penicillin-Streptomycin (100×)	Gibco	Cat# 15140122
Phosphate buffered saline 1×	Sigma-Aldrich	P3813
Poly-L-Ornithine	Sigma-Aldrich	P4957
Prolong Gold Antifade	Abcam	Ab104135
Saline (NaCL)	Braun	Cat# 190/12606090/0713
Sodium Bicarbonate	Sigma-Aldrich	S5761
Triton-X 100	Sigma-Aldrich	Cat# 9002-93-1
Trypan Blue	Invitrogen	T10282
Trypsin – EDTA 0.25%	Sigma-Aldrich	T4049

**Experimental models: Organisms/strains**		

Sprague Dawley rats, Adult (10 weeks), Female	Janvier	N/A

**Other**		

15 mL conical tube	Corning	Cat# 430766
2D variable speed rocker	VWR	Cat# 75832-308
50 mL conical tube	Corning	Cat# 430291
Accu-jet Pro Motorized pipette controller	Sigma-Aldrich Scientific	Z637645-1EA
ART 10 Barrier Pipette Tips	Thermo Fisher Scientific	Cat# 2039-HR
ART 200 REACH Barrier Pipette Tips	Thermo Fisher Scientific	Cat# 2069-HR
ART 1000 REACH Barrier Pipette Tips	Thermo Fisher Scientific	Cat# 2079-HR
Bench Safe absorbent spillage protection	Sirane	BES60
BioWizard Golden GL-130 Class II Laminar Flow hood	Kojair	Cat# 021464
Bone scissors Beebee sharp (curved)	AgnTho’s	Cat# 03-745-105
Countess cell counting chamber slide	Invitrogen	C10283
Countess™ II Automated Cell Counter	Invitrogen	AMQAX1000
Cover glass round Menzel Gläser	Thermo Scientific	Cat# 630-1846
Disposable graduated transfer pipettes	VWR	Cat# 612-4545
Dissecting scissors	AgnTho’s	Cat# 03-373-100SB
Dumont #5 Forceps	AgnTho’s	Cat# 11251-10
Dumont 5/90 forceps	AgnTho’s	Cat# 11253-29
Eppendorf 2 mL safe-lock tubes (colorless)	Eppendorf	Cat# 0030123620
Eppendorf 2 mL safe-lock tubes (light protection)	Eppendorf	Cat# 0030120248
EVOS M5000	Thermo Fisher Scientific	AMF5000
Finnpipette F2 p10	Thermo Fisher Scientific	Cat# 1184850 (Kit 2)
Finnpipette F2 p20	Thermo Fisher Scientific	Cat# 1184850 (Kit 2)
Finnpipette F2 p200	Thermo Fisher Scientific	Cat# 1184850 (Kit 2)
Finnpipette F2 p1000	Thermo Fisher Scientific	Cat# 1184850 (Kit 2)
Heidemann spatula	AgnTho’s	Cat# 85-111-002
Hippocampal dissection tool	AgnTho’s	Cat# 10099-15
Ice box (Styrofoam)	N/A	N/A
IKA MS 3 Basic Vortex Mixer	Fisher Scientific	Cat# 10492342
Isoflurane Vaporizer Std Fill 5% CM	Vet-Tech	AN003
Microcentrifuge tube (0.5 mL)	Eppendorf	Cat# 0030 121.503
Microcentrifuge tube (1.5 mL)	Eppendorf	Cat# 0030 120.086
NUNC Cryotubes	Merck	V7509
Optima T100 Heated Circulating Bath	Grant Instruments	T100-ST12
Paper towels (optional)	N/A	N/A
Pencil	N/A	N/A
Poly-L-Ornithine Solution (0.01%)	Sigma-Aldrich	A-004-C
Razor Blade (double-sided blade, single use)	N/A	N/A
Rodent Guillotine	World Precision instruments	DCAP-M
Rongeurs (Luer, curved)	AgnTho’s	Cat# 31-377-150
Rotina 420R Centrifuge	Hettich Zentrifugen	Cat# 4701
Serological pipette (5 mL)	Corning	Cat# 4487
Serological pipette (10 mL)	Corning	Cat# 4488
Serological pipette (25 mL)	Corning	Cat# 4489
Sharp/Blunt surgical scissors	AgnTho’s	Cat# 03-022-155
Steri-Cycle CO2 Incubator	Thermo Fisher Scientific	Cat# 317
Sterile non-tissue culture treated 6-well plate	Thermo Fisher Scientific	Cat# 150239
Sterile tissue culture treated 6-well plate	VWR	Cat# 10861-696
Sterile OP-Towel	BARRIER	Cat# 800430
Superfrost Plus Coverslides	VWR	Cat# 631-9483
Vannas spring scissors	AgnTho’s	Cat# 05-220-085
Waste bag	N/A	N/A
Wheaton Coplin staining jar (Lid)	Merck	S6016
ZEISS Axio Vert A1	Carl Zeiss Microscopy	ZEISS Axio Vert.A1

## Materials and equipment

***Alternatives:*** A 4% Paraformaldehyde solution can be used as alternative, but the glyoxal solution produces better quality staining for targets in this protocol.

**CRITICAL:** Glyoxal is a cytostatic and should only be handled inside a laminar flow cabinet while the user wears gloves and long-sleeved protective gear/lab coats. Discard and replace protective gear immediately in case of spills.

## Step-by-step method details

### Main procedure: Dissection

**Timing: 10 min**

This step describes the procedure for dissecting out each of the three neurogenic niches (SVZ, SGZ and CC) containing the adult neural stem cells. It is **not** advisable to extract more than one neurogenic niche per animal as keeping tissue over prolonged time periods on ice will significantly impact the percentage of retrieved viable cells. To mitigate this, additional workstations can be set up to process each extracted niche in parallel.

**CRITICAL:** The entire process from decapitation to transport should take **no longer than 5 min**. It is recommended to familiarize with the procedure practicing on cadavers before attempting the first harvest. Try to avoid pieces of white matter being mixed in during dissection (especially the CC), as these will significantly hamper the dissociation. Troubleshooting 1 may be useful.

Brain Dissection:

1. Euthanize the animal by first inducing deep anesthesia with 4% Isoflurane followed by decapitation. Disinfect the animal thoroughly before starting the dissection.
2. Remove head and cut open the scalp with surgical scissors to expose the skull.
3. Cut off the remnant of the spinal column with the bone scissors. Break open the back of the skull with rongeurs and cut away the pieces of skull with the bone scissors.
4. Insert the sharp blade of the surgical scissors into the skull along the longitudinal fissure and cut open the skull (superficial damage to the brain during this step is not a problem).
5. Remove the surgical scissors and carefully insert the Heidemann spatula through the crack down the midline and release the Dura Mater from the skull on both sides before opening the skull with the rongeurs.
6. When fully exposed, extract the brain with a curved hippocampal dissection tool and transfer to the ice-cold glass plate, and soak in ice-cold sterile saline.
7. Discard remains and surgical barrier sheet.

**Figure 2:**
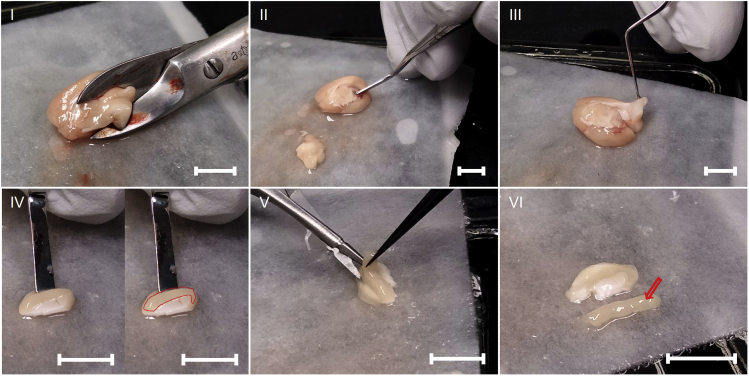
Dissection of Dentate Gyrus for extraction of SGZ-derived NSCs (I) Removal of the remaining part of the brain stem, cerebellum, and largest part of the basal ganglia, exposing the hippocampus on one hemisphere. (II) Cutting the hippocampus in two equal parts down the middle (Dorsal-Ventral axis). (III) Extraction of the dorsal hippocampal section. (IV) (left) Location of the dentate gyrus within the hippocampal formation, (right) dentate gyrus highlighted. (V) Extraction of the dentate gyrus from the hippocampal formation. (VI) Dentate gyrus (arrow) after extraction from the hippocampal formation (above), all scale bars: ≈5.0 mm.

For subgranular zone (SGZ) extraction (Figure 2):

**CRITICAL:** Make sure the tissue remains chilled at all times by periodically dousing it in ice-cold sterile saline to improve cell survival.

8. Remove the cerebellum entirely before separating the two hemispheres sagittally down the midline using a sterilized and chilled razor blade.
9. Remove the remaining part of the brain stem, cerebellum and as much of the basal ganglia as possible with the bone scissors, exposing the hippocampus on one hemisphere.
10. Using the Heidemann spatula, cut the hippocampus in two equal parts down the middle (dorsal-ventral).
11. Gently move the Heidemann spatula underneath the dorsal hippocampal section and gently flip it out of the brain and onto the filter paper-covered chilled glass plate. Discard what is left of the hemisphere.
12. Using the Heidemann spatula and forceps, isolate the dentate gyrus from the rest of the hippocampal formation.
13. Discard the CA regions of the hippocampus, (unless there is capacity and set up enabling simultaneous processing of the relevant tissue), remove the filter paper and thoroughly dice the dentate gyrus tissue with a chilled and sterile razor blade, and transfer to the vial with ice-cold Neurobasal 1× with a graduated transfer pipette for transport and dissociation.
14. Repeat for 2^nd^ hemisphere, and transport tissue from both hemispheres in the same 15 mL tube on ice.

**Figure 3:**
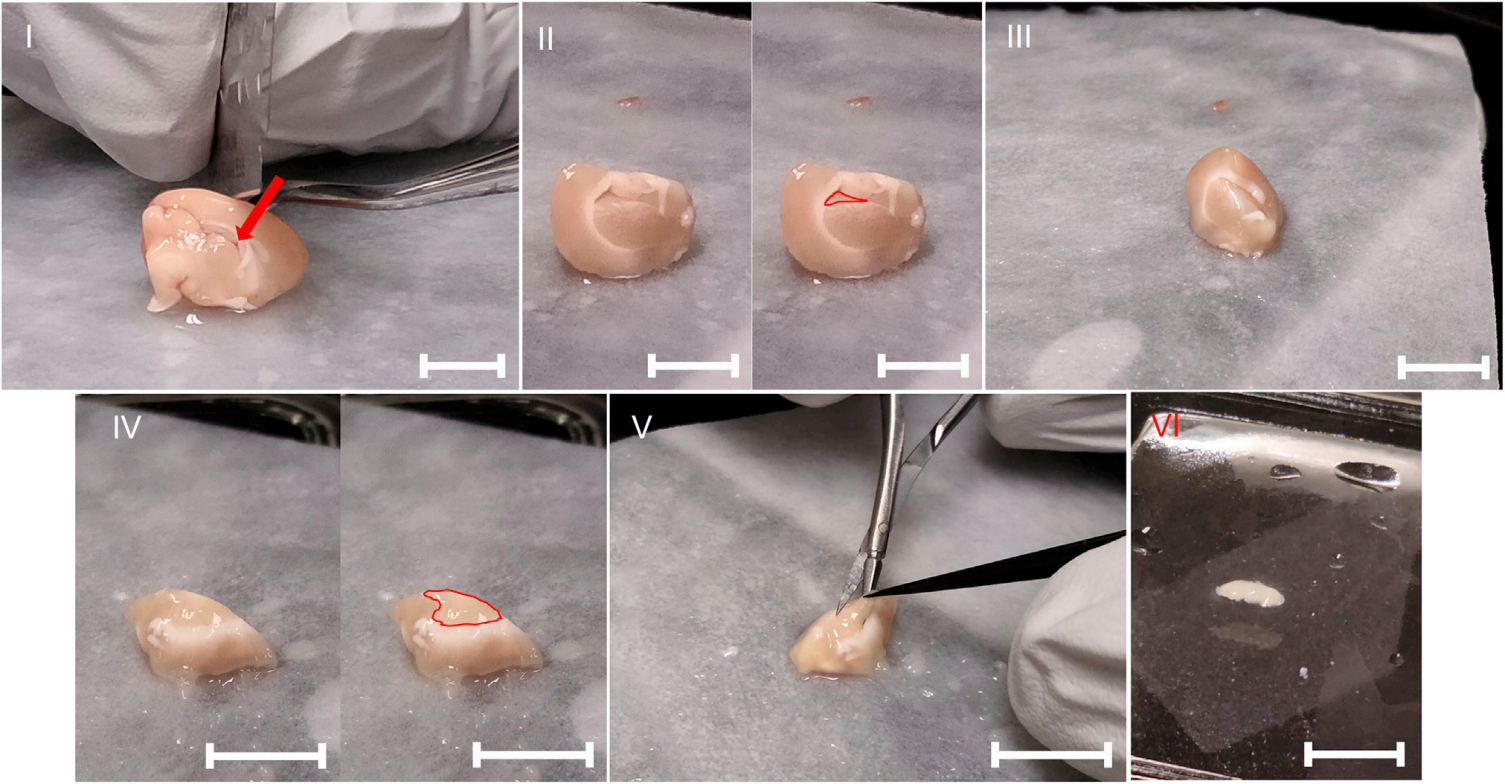
Dissection of Subventricular zone for extraction of SVZ-derived NSCs (I) Location of hippocampal dorsal apex (arrow) as landmark. (II) (left) location of lateral SVZ as seen after cutting the brain coronally, (right) lateral SVZ highlighted. (III) View of lateral SVZ after removal of basal ganglia and medial SVZ. (IV) (left) lateral SVZ fully exposed after removal of overhanging cortical and corpus callosum tissue, (right) lateral exposed SVZ highlighted. (V) Extraction of lateral SVZ. (VI) SVZ after extraction, all scale bars: ≈5.0 mm.

For subventricular zone (SVZ) extraction (Figure 3):

**CRITICAL:** Make sure the tissue remains chilled at all times by periodically dousing it in ice-cold sterile saline to improve cell survival.

15. Remove the cerebellum entirely before separating the two hemispheres sagittally down the midline using a sterilized and chilled razor blade.
16. Using the apical tip of the hippocampus as a marker, cut one hemisphere coronally 0.5 mm anterior to the hippocampal apex.
17. Discard the caudal part of the brain hemisphere and trim away remnant of basal ganglia and cut away the overhanging cortex using the fine forceps and small dissection scissors, fully exposing the Subventricular zone.
18. Using the Vannas spring scissors and forceps, gently cut out the anterior 2/3 of the lateral SVZ in a single thin sheet.

***Note:*** Make sure the tissue excised is a uniform light brown color. White webbing-like tissue will indicate when too much tissue has been cut out laterally.

***Note:*** The rate of NSC proliferation has been shown to occur in the anterior SVZ, so to reduce the amount of additional tissue, it is recommended to only extract the anterior 2/3 (Falcão et al., 2012). The lateral wall of the SVZ mainly gives rise to neurons and astrocytes, while the medial wall primarily gives rise to oligodendrocytes (Mizrak et al., 2019), it is recommended to take this into consideration when dissecting.

19. Transfer the SVZ onto the filter paper-covered chilled glass plate and dice thoroughly with a sterile and chilled razor blade, and transfer to the vial with ice-cold Neurobasal 1× with a graduated transfer pipette for transport and dissociation.
20. Repeat for 2^nd^ hemisphere, and transport tissue from both hemispheres in the same 15 mL tube on ice.

Extracting SGZ & SVZ neurogenic niches from the same animal.

**CRITICAL:** Make sure tissue dissociation steps (39–53) are set up in such a way that the tissues can be processed in parallel. It is recommended to have two parallel setups (fume hood and centrifuge) with one person processing each extracted niche separately. It is not recommended to additionally extract the CC neurogenic niche from the same animal as spinal cord dissection and CC extraction take a long time and this will negatively impact cell viability.

21. Prepare the brain for the niche extractions as described in steps 8–10 & 15–17. Ensure that all pieces of the brain are continuously chilled with ice-cold PBS throughout the procedure to minimize cell loss.
22. Extract the SGZ as described in steps 10–14 (Figure 2).
23. Immediately extract the SVZ as described in steps 17–20, excluding the removal of the caudal section of the hemisphere if the SVZ is extracted prior to the SGZ (Figure 3).
24. Proceed to tissue dissociation section.

Spinal cord dissection:

25. Open up chest and stomach, removing all internal organs using the surgical scissors.
26. Cut through the spinal column at the base of the animal’s neck using the bone scissors.
27. Release the skin of the back from the spinal column and ribs all the way down to the hips of the animal and cut through the spinal column just rostral to the Rectus Femoris muscles using the bone scissors.
28. Insert one blade of the dissection scissors into the spinal column and carefully cut both sides of each vertebra to expose the spinal cord.

***Note:*** Carefully cut 1 vertebra at a time, always pointing the tip of the dissection scissors outwards, away from the spinal cord as damaging the spinal cord will make later steps much more difficult.

29. Quickly douse the exposed spinal cord with ice-cold sterile saline before extraction.
30. Carefully grip the rostral tip of the spinal cord with a pair of fine forceps (non-dominant hand) and lift gently until resistance is met. Carefully cut the root ganglia on either side stepwise with a pair of small dissection scissors (dominant hand) while gently lifting the spinal cord as it releases.
31. Cut the spinal cord right below the lumbar enlargement and transfer to the filter paper-covered chilled glass plate and douse again with ice-cold sterile saline.
32. Discard remains and surgical barrier sheet.

**Figure 4:**
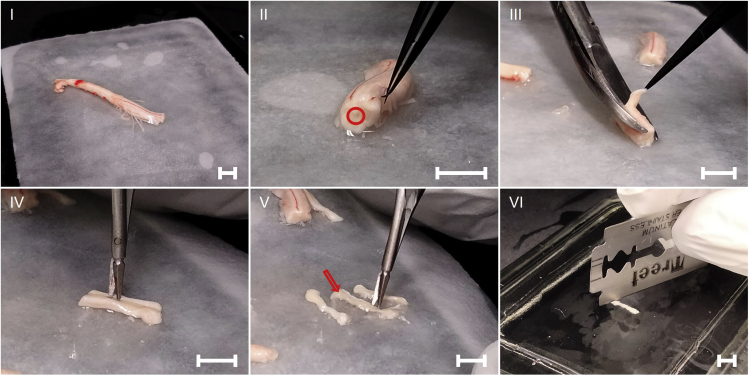
Dissection of spinal cord for extraction of CC-derived NSCs (I) Extracted spinal cord. (II) Location of CC neurogenic niche within the spinal cord. (III) Removal of lateral white and gray matter from spinal cord. (IV) Isolation of CC and small amount of surrounding tissue from white and gray matter of the spinal cord. (V) Isolated CC (arrow). (VI) Dicing of CC tissue in preparation for dissociation for NSC isolation, same procedure for SVZ and SGZ tissues, all scale bars: ≈5.0 mm.

For central canal (CC) extraction (Figure 4):

**CRITICAL:** Make sure the tissue remains chilled at all times by periodically dousing it in ice-cold sterile saline to improve cell survival.

33. Cut the spinal cord into 2–3 1 cm long pieces using a chilled and sterile razor blade.

***Note:*** Rest the spinal cord against the side of the forceps and let the razor blade gently cut through the spinal cord against the forceps. Do not compress the spinal cord as it will make later steps more difficult.

34. Using the bone scissors, gently cut of the white matter on either side of the piece of spinal cord (lateral sides, identify the dorsal side of the spinal cord by the large blood vein running down the midline).
35. Cut the white matter off the top of the spinal cord.

***Note:*** During these steps it is important to maintain orientation of the spinal cord and precision when cutting as the target area is very small and it can very quickly become difficult to see where to cut.

36. Grab the gray matter containing the central canal with the fine forceps and carefully cut it off from the remaining ventral white matter of the spinal cord piece.
37. Remove filter paper and place the piece of gray matter onto a clean part of the glass plate and dice thoroughly with a sterile and chilled razor blade, and transfer to the vial with ice-cold Neurobasal 1× with a graduated transfer pipette for transport and dissociation.
38. Repeat the procedure for the remaining spinal cord sections (start with 1–2, expand to 2–3 when more experienced with the procedure), and transport tissue from all sections in the same 15 mL tube on ice.

**CRITICAL:** Make sure to clean the extracted tissue off white matter during the dissection. Pieces of white matter do not dissociate as easily and resulting clumps may get stuck in the pipette tip when aspirating. This will narrow the opening of the pipette tip, effectively increasing the pressure exerted on the cells during aspiration, resulting in a drastic reduction of cell survival.

### Main procedure: Tissue dissociation

**Timing: 45 min**

This step describes the process of dissociating the tissue extracted through chemical digestion and mechanical dissociation. This process isolates the adult NSCs into a suspended single cell solution and initiates their expansion. Begin this step immediately after each dissection. Troubleshooting 1 may be useful. Adapted for use with all three other neurogenic niches from Dizon et al. (Dizon et al., 2006).

39. Spin down the tissue at 100× g for 1 min at RT (20°C–23°C).
40. In a laminar flow cabinet, aspirate the transport media and resuspend the tissue in the same 15 mL tube using 5 mL ice-cold HBSS −/− using a serological pipette. Spin down the tissue at 100 × *g* for 1 min at RT (20°C–23°C).

***Note:*** To avoid loss of tissue at this stage the pellet should ideally be broken up using the force of the stream of the HBSS being added rather than through aspirations, as the tissue will tend to stick to any pipette tips and serological pipettes.

41. Repeat steps 39–40 once.
42. Aspirate the HBSS and resuspend the tissue from 1 animal in 1 mL accutase for dissociating the CC -derived, SVZ -derived tissue or 1 mL trypsin for dissociating the SGZ-derived tissue (this should be at least at RT (20°C–23°C) when being added), breaking up the pellet with the stream of liquid.

**Figure 5:**
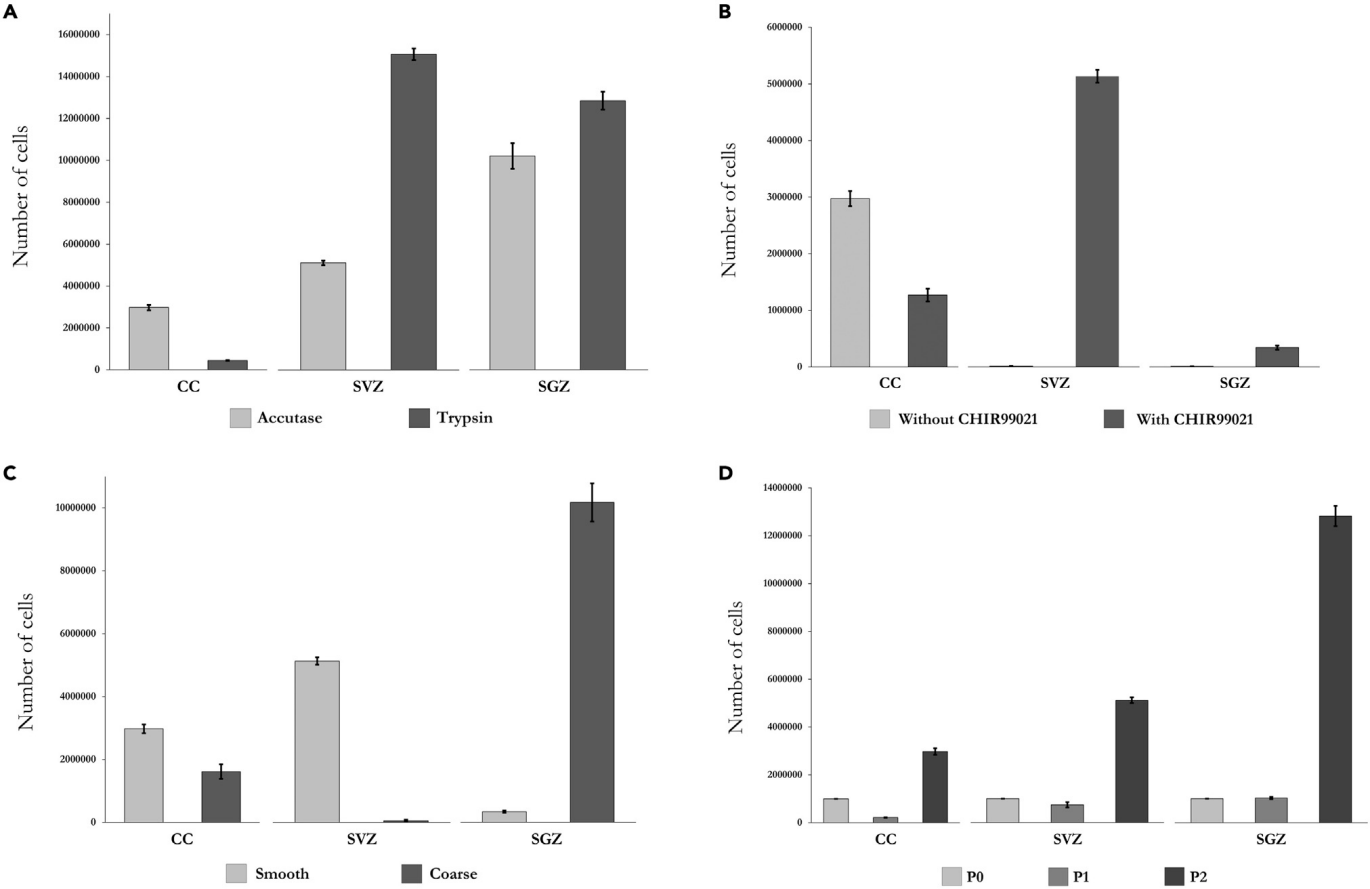
Representative numbers of live cells obtained from CC, SVZ and SGZ-derived NSC expansion (A) Number of live CC, SVZ and SGZ-derived cells at P2 when dissociated with accutase or trypsin. (B) Effect of 10 μM CHIR 99021 of survival/proliferation of CC (see also Figures 9A–9D), SVZ and SGZ-derived NSCs (all cultured on smooth surface topography). (C) Effect of culture vessel surface topography on survival and proliferation of CC, SVZ and SGZ-derived NSCs (P2). Note: all 3 neurogenic niches dissociated with accutase. (D) Expansion of CC, SVZ and SGZ-derived NSC cultures over 20 days (P0-P2). Note: SGZ tissue dissociated with trypsin. All cell counts are derived from representative experiments for each condition.

**CRITICAL:** Both accutase and trypsin can be used for SVZ and SGZ dissociation, and the expected percentage of surviving cells directly following dissection and dissociation prior to proliferation is shown in Table 5. Trypsin will increase proliferative potential in both compared to accutase (see Figure 5A for representative number of cells after 20 days of proliferation) but may likely result in increased spontaneous glial-fated differentiation of SVZ-derived cells (see expected outcome: SVZ). However, only accutase should be used for dissociating the CC-derived tissue as trypsin will severely impact NSC yields from this neurogenic niche (Figure 5A).

43. Incubate the tissue for 15 min (accutase) or 5 min (trypsin) in an incubator at 37°C. Following incubation, mechanically dissociate the tissue by repeatedly triturating the solution 500× using a p100 or p200 pipette set to 100 μL.

**Figure 6:**
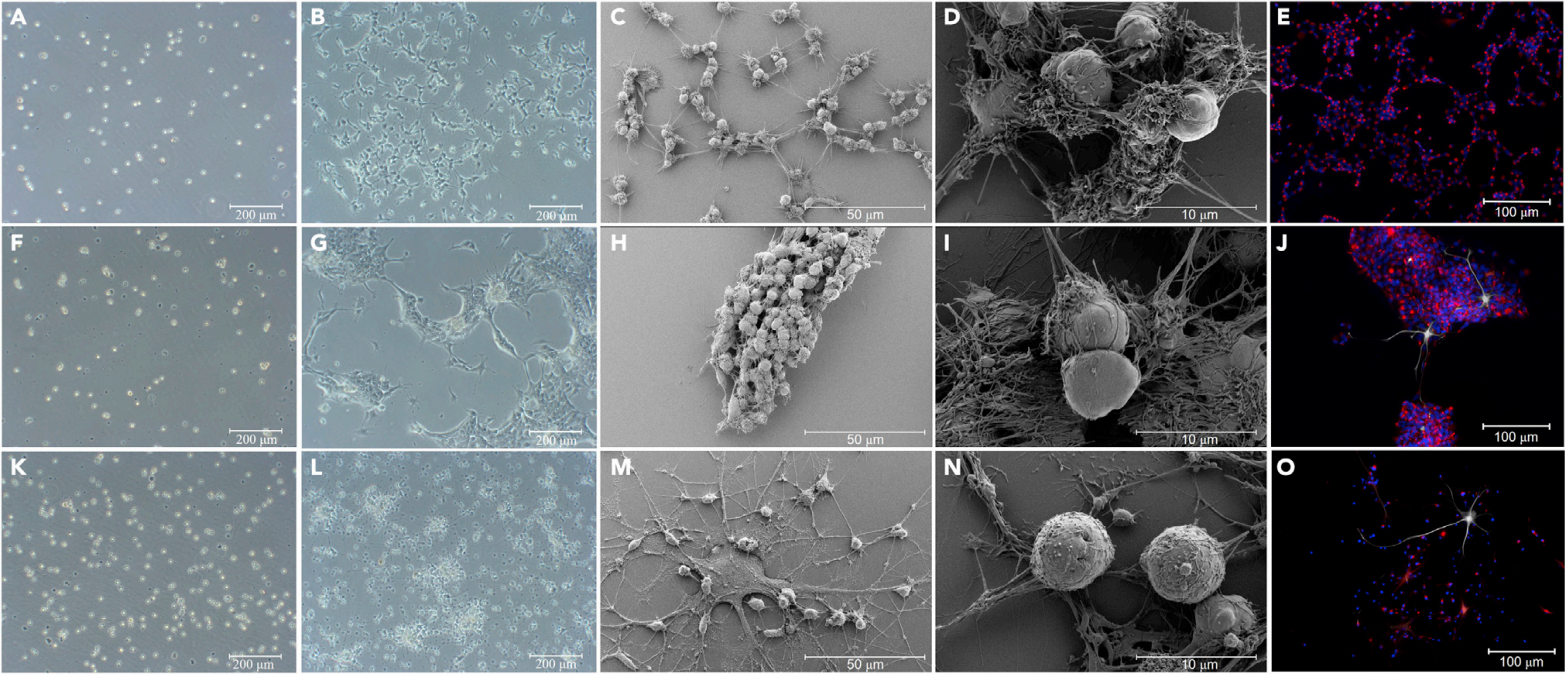
Development and morphological characterization of CC, SVZ and SGZ-derived NSCs (A) Phase contrast of CC-derived NSCs directly following P1 passage (scare bar: 200 μm). (B) Phase contrast of CC-derived NSCs 3 days post P2 passage, seeded onto PLO-Laminin coated coverslips (scare bar: 200 μm). (C) SEM of CC-derived NSCs 3 days post P2 passage, seeded onto PLO-Laminin coated coverslips (scale bar: 50 μm). (D) SEM of CC-derived NSCs 3 days post P2 passage, seeded onto PLO-Laminin coated coverslips (scale bar: 10 μm). (E) Immunostaining of CC-derived NSCs 3 days post P2 passage, seeded onto PLO-Laminin coated (scale bar: 100 μm). (F) Phase contrast of SVZ-derived NSCs directly following P1 passage (scare bar: 200 μm). (G) Phase contrast of SVZ-derived NSCs 3 days post P2 passage, seeded onto PLO-Laminin coated coverslips (scare bar: 200 μm). (H) SEM of SVZ-derived NSCs 3 days post P2 passage, seeded onto PLO-Laminin coated coverslips (scale bar: 50 μm). (I) SEM of SVZ-derived NSCs 3 days post P2 passage, seeded onto PLO-Laminin coated coverslips (scale bar: 10 μm). (J) Immunostaining of SVZ-derived NSCs 3 days post P2 passage, seeded onto PLO-Laminin coated coverslips (scale bar: 100 μm). (K) Phase contrast of SGZ-derived NSCs directly following P1 passage (scare bar: 200 μm). (L) Phase contrast of SGZ-derived NSCs 3 days post P2 passage, seeded onto PLO-Laminin coated coverslips (scare bar: 200 μm). (M) SEM of SGZ-derived NSCs 3 days post P2 passage, seeded onto PLO-Laminin coated coverslips (scale bar: 50 μm). (N) SEM of SGZ-derived NSCs 3 days post P2 passage, seeded onto PLO-Laminin coated coverslips (scale bar: 10 μm). (O) Immunostaining of SGZ-derived NSCs 3 days post P2 passage, seeded onto PLO-Laminin coated coverslips (scale bar: 100 μm. I&J&O Blue: Hoechst, White: GFAP (Rabbit), Red: Nestin).

**Figure 7:**
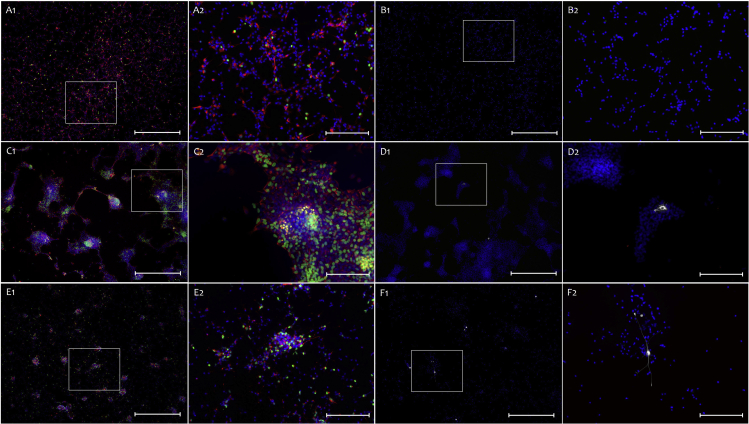
Characterization of CC, SVZ and SGZ-derived NSCs (A and B) NSC marker characterization of CC-derived cells (scale bar 1: 200 μm, 2: 50 μm). (C and D) NSC marker characterization of SVZ-derived cells (scale bar 1: 200 μm, 2: 50 μm). (E and F) NSC marker characterization of SGZ-derived cells (scale bar 1: 200 μm, 2: 50 μm). (A&C&E Blue: Hoechst, Green: EdU, Yellow: SOX2, Red: Nestin. B&D&F Blue: Hoechst, Yellow: DIx2, White: GFAP (Chicken), Red: A2B5). All cells cultured in appropriate culture vessels for expansion.

**Figure 8:**
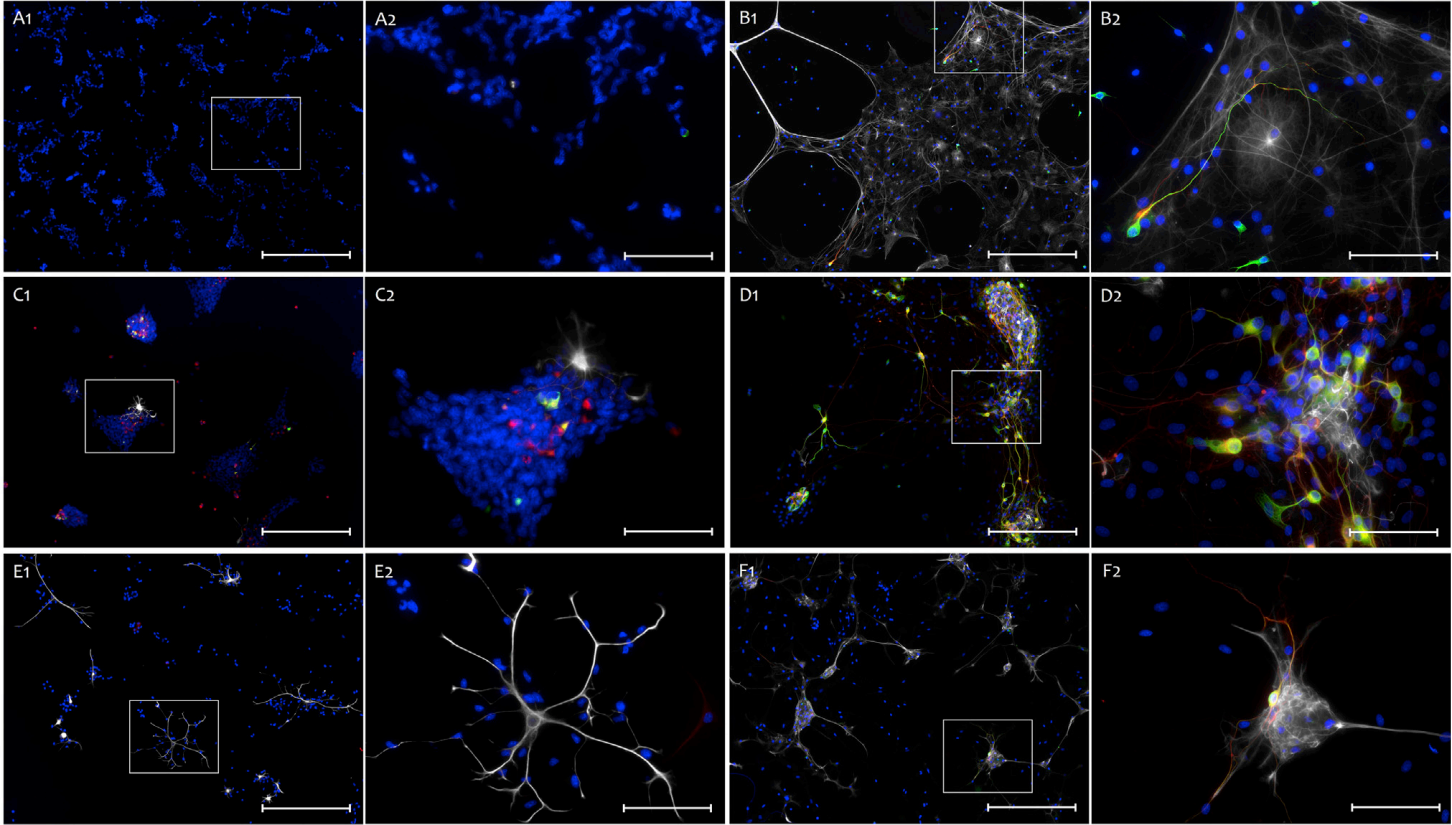
CC, SVZ and SGZ-derived NSC neural lineage differentiation (A and B) Neural lineage differentiation potential of CC-derived NSCs at start of differentiation (A), and after 30 days of differentiation (B) (scale bar 1: 200 μm, 2: 50 μm). (C and D) Neural lineage differentiation potential of SVZ-derived NSCs at start of differentiation (C), and after 30 days of differentiation (D)(scale bar 1: 200 μm, 2: 50 μm). (E and F) Neural lineage differentiation potential of SGZ-derived NSCs at start of differentiation (E), and after 30 days of differentiation (F) (scale bar 1: 200 μm, 2: 50 μm). A-F Blue: Hoechst, Green: Map2, White: GFAP (Rabbit), Red: Beta-3-Tubulin. All cells cultured on 13 mm PLO-Laminin coated coverslips .

**Figure 9:**
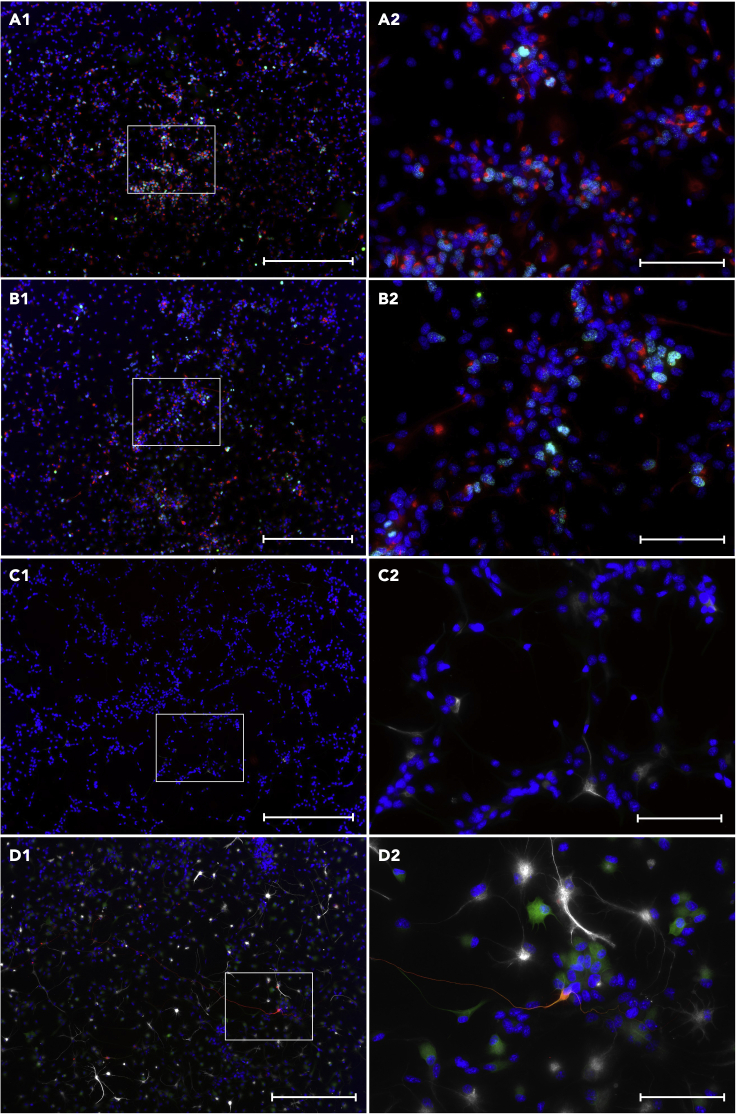
Effect of CHIR 99021 treatment of CC-derived NSCs (A and B) Effect CHIR 99021 absent (A) or present (B) in CC-derived NSCs after 6 days of expansion (Blue: Hoechst, Green: EdU, Red: Nestin, scale bar 1: 1250 μm, 2: 100 μm). (C and D) Auto-differentiation of CC-derived NSCs with CHIR 99021 absent (C) or present (D) after 6 days of expansion (Blue: Hoechst, Green: Map2, White: GFAP (Rabbit), Red: Beta-3-Tubulin,scale bar 1: 1250 μm, 2: 100 μm).

**Figure 10:**
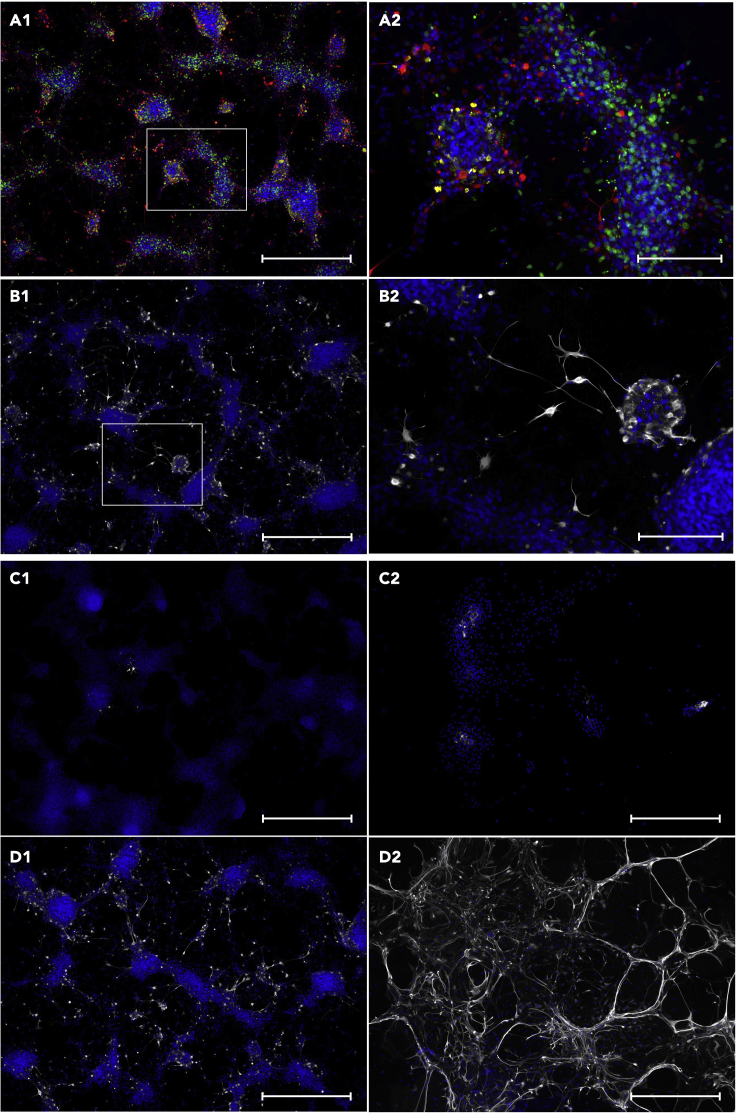
Effects of trypsin dissociation on SVZ-derived NSCs (A) NSC marker characterization of trypsin-dissociated SVZ-derived NSCs (Blue: Hoechst, Green: EdU, Yellow: SOX2, Red: Nestin, scale bar 1: 1250 μm, 2: 100 μm). (B) NSC marker characterization of trypsin-dissociated SVZ-derived NSCs (Blue: Hoechst, Yellow: DIx2, White: GFAP (Chicken), Red: A2B5), scale bar 1: 1250 μm, 2: 100 μm). (C) GFAP (white) positive cells in accutase-dissociated NSC culture at start of differentiation (C-1) and after 30 days of differentiation (C-2) (scale bar: 1250 μm). (D) GFAP (white) positive cells in trypsin-dissociated NSC culture at start of differentiation (D-1) and after 30 days of differentiation (D2), (scale bar: 1250 μm).

***Note:*** Avoid introducing bubbles during this step, to avoid this as well as shearing forces that could reduce viability if the pipette tip touches the very bottom of the tube while aspirating. The tip can be stabilized at the right height by holding the shaft of the pipette at the opening of the 15 mL tube with the same hand holding the tube.

44. Transfer the dissociated tissue into a new 15 mL tube already containing 1 mL warm wash media.
  a. Wash the lower part of the inside of the original tube using 1 mL warm Neurobasal 1× wash media and add this to the new tube to recover as many cells as possible.
  b. Add another 2 mL of Neurobasal 1× wash media for a final volume of 5 mL.

***Note:*** Do the washing of the old tube using the same tip used to transfer the cells to minimize loss of cells.

45. Spin down the cells at 150 × *g* for 5 min at RT (20°C–23°C).
46. Aspirate the washing media and resuspend the pellet in 4 mL warm wash media and spin down the cells again at 150 × *g* for 5 min at RT (20°C–23°C).

***Note:*** For all washing steps, the cell pellet should be broken up using only the stream of media from a p1000 pipette set to 1000 μL, if at all possible, as the loss of cells tends to be substantial if repeated triturations are required, as they will stick to the plastic of the pipette tip.

47. Repeat steps 45–46 once.
48. During the wash steps, prepare a non-tissue culture-treated 6-well plate for seeding by adding 3.5 mL of culturing media to 1–3 wells and place the plate in a humidified incubator (5% CO_2_, 37°C). Recommended seeding density is 5 × 10^5^/well. Also, keep in mind that due to proliferation the number of wells should be doubled at P1.
49. Following the second wash, remove the wash media and resuspend the pellet in 1 mL culturing media.

**CRITICAL:** Carry out the next steps quickly to ensure cell viability does not decrease.

50. Take 10 μL of resuspended cell solution and add this to 10 μL of 0.4% Trypan Blue in a 500 μL Eppendorf. Mix gently but thoroughly by triturating 10× times and apply 10 μL to both sides of a cell counting chamber slide.
51. Count the cells using an automatic cell counter (or other method of choice) and record the numbers from both sides of the chip. From the average of the two counts of live cells, determine the volume containing 5 × 10^5^ cells.

**Calculation:** Average of total number of live cells ÷ 1000 for number of live cells per μL of cell solution. 5 × 10^5^ ÷ number of live cells/μL gives how many μL of cell solution contain 5 × 10^5^ live cells.

52. Add the volume of cell suspension containing 5 × 10^5^ live cells to each well in the 6-well plate prepared in step 51.
53. Place the plate in a humidified incubator (5% CO_2_, 37°C) and leave undisturbed for 4 days.

***Note:*** For best results make sure that cells are evenly distributed throughout the wells. One option here is to gently swirl the plate, switching between clockwise and counterclockwise.

**CRITICAL:** Ensure that the tissue has been dissociated properly before performing cell counts, to obtain appropriate estimates of viable cells. This is particularly important for the CC dissociation as up to 90% of the live cells will die within the first few days leaving only the NSCs behind.

***Note:*** Presence of debris/dying cell will not notably impact NSC behavior but should be cleared away by washing the neurospheres during media changes and passages. Troubleshooting 2 may be useful.

### Main procedure: Media change

**Timing: 1–3 h (depending on number of cultures)**

This stage describes the media changes, a critical main part of the maintenance process during the expansion phase of the experiment. Troubleshooting 3 may be useful if problems with cell proliferation are encountered.

**CRITICAL:** In this protocol CHIR 99021 is used to prevent spontaneous differentiation (and subsequent cell death during dissociation) and increase proliferation in the SVZ-derived NSCs **when grown as monolayer cultures** (Pachenari et al., 2017; Tang et al., 2017). CHIR 99021 is not required for suspended NSC cultures as demonstrated by most other protocols. **Do not** add CHIR 99021 to CC-derived NSC cultures as this will induce differentiation (see expected outcomes: CC). Paradoxically, adding CHIR 99021 to late-stage differentiating CC NSCs may induce de-differentiation to a more NSC/progenitor-like state and have been used for cell-to-stem cell reprogramming (Han et al., 2016; Liu et al., 2018; Tian et al., 2018; Zhu et al., 2014). CHIR 99021 is also critical for cell survival of the SGZ-derived NSCs monolayer cultures using **this protocol** (Figure 5B), however troubleshooting 6 may also improve SGZ NSC viability beyond what is presented in this protocol.

**CRITICAL:** During P0 NSCs may form adherent cultures on the well bottom, and after P1 passage, all NSCs should form an adherent monolayer prior to the 1^st^ media change. This will allow more cells to access nutrients, avoiding cell death from suffocation within neurospheres with minimal impact on NSC characteristics and behavior (Babu et al., 2007; Conti and Cattaneo 2010; Miyazaki et al., 2012; Mori et al., 2006; Pollard et al., 2006; Zheng et al., 2006).

**CRITICAL:** Surface topography of culture vessel is of high importance to monolayer NSC survival and proliferation: The SVZ-derived NSCs will spontaneously differentiate and die on any surface that is not completely smooth (Example: non-tissue culture treated plates, key resources table). This can be mitigated to some degree by seeding NSCs on glass coverslips coated with Poly-L-Ornithine and Laminin solution (Figure 5C). The SGZ-derived NSC monolayer cultures thrive best when they have a rough physical environment to attach to, drastically improving rate of proliferation (Example: tissue culture treated plates, key resources table), however, survival and proliferation is impacted on both coated and non-coated glass coverslips (Figure 5C). The CC-derived NSCs are much more versatile in terms of surface topography and will thrive on both smooth and non-smooth surfaces, including coated and uncoated glass coverslips, however, smooth surfaces provide the highest rate of proliferation (Figure 5C). Cell seeding density have also been shown to have an effect on NSC behavior on different culture vessel topographies (Cha et al., 2017).

54. Leave the cells in the incubator for 4 days to expand following the dissociation before the 1^st^ media change.
55. Prepare media according to Tables 2, 3, and 4 and pre-warm to 37°C in a water bath.
56. Using a 5 mL serological pipette, remove cell media from 1 well, triturate 2–3 times to wash off any adherent cells before transferring the media to an empty 15 mL tube. Triturate cell-containing media quickly 3–4 times to clean the neurospheres of any adherent cell debris.

**CRITICAL:** Avoid aspirating in air as that will damage and potentially kill cells.

57. Pipette 1 mL of fresh media into the well quickly, making sure any remaining cells are submerged, using the same 5 mL serological pipette as during step 56.
58. Repeat the procedure for remaining wells on the same plate.
59. Centrifuge the tube containing the cells at 150 × *g* for 5 min at room temperature.
60. Remove supernatant and replace with 2 mL fresh media per well. Using a p1000 pipette, triturate 3–4 times to break up the pellet and return cells to their wells.

***Note:*** Triturate twice before reseeding each well, as the cells sink quickly, to maintain equal number of cells between the wells (there may be more cells in the last well reseeded if this is not done properly prior to seeding). To avoid bias in later cell counts all wells seeded from the same initial population need to be pooled and counted as one experimental unit for an accurate record of cell numbers and rate of proliferation.

61. Return plate to incubator.

**Suggested schedule:** P0: Harvest cells on day 0, change media on days 4 and 7, passage to P1 on day 10. P1: Change media on days 13, 16 and 18 and passage to P2 on day 20.

***Note:*** For P1 and later generations: Keep an eye on media acidification (phenol red shifting to a yellow hue) as growth rate may require media changes more often (P2 may require changing every 18–12 h if expansion is continued beyond 20 DIV as rate of proliferation increases exponentially).

### Main procedure: Passaging

**Timing: 45–60 min (per passage)**

This step describes the passaging of cultured NSCs for the purpose of breaking up suspended neurospheres and adherent monolayer colonies into single cell suspension to be reseeded for redistribution and avoiding cell death from being suffocating within the core of neurospheres and overgrown adherent 3D structures. If NSCs do not properly form monolayers after P1, troubleshooting 4 may be useful.

If there are suspended cells/neurospheres (relevant for P1 passage):

62. Remove media with cells using a 5 mL serological pipette and transfer to empty 15 mL tube.
63. Very carefully add 1 mL warm (37°C) Neurobasal 1× to the well to keep the attached cells hydrated, make sure not to dislodge any adherent cells when doing so.
64. Centrifuge tube with cells at 150 × *g* for 5 min at room temperature.

 Aspirate supernatant and resuspend cells in dissociation agent (accutase/trypsin, 1 mL per well).

65. Carefully remove the hydration media from each well, take care not to detach any adherent cells.
66. Triturate dissociation agent with cells twice, aspirate and return 1 mL to each well and continue from step 68.

If there are no suspended cells/neurospheres:

67. Gently remove cell media and replace with 1 mL dissociation agent per well.

Continued for both:

68. Incubate the cells for 15 min (accutase) or 5 min (trypsin) in an incubator (5% CO_2_, 37°C). Following incubation, aspirate off the dissociation agent, and use it to wash the bottom of the well, collecting the cells in the bottom. Transfer all cells and dissociating agent to a 15 mL tube and mechanically dissociate the cells by triturating the solution 100× times using a p200 pipette for volumes =< 2 mL, or p1000 pipette for volumes > 2 mL, triturating approximately 20% of the total volume of liquid in the vial each time.

***Note:*** Avoid introducing bubbles during this step, to avoid this as well as shearing forces that could reduce viability if the pipette tip touches the very bottom of the tube while triturating. The tip can be stabilized at the right height by holding the shaft of the pipette at the opening of the 15 mL tube with the same hand holding the tube.

69. Add 4 mL of warm Neurobasal 1× wash media to the tube (for 1 mL dissociating agent. Add 1 additional mL of wash media for every additional 1 mL of dissociation agent) to neutralize and wash out the dissociation agent (see troubleshooting 1 for issues when using trypsin).
70. Spin down the cells at 150 × *g* for 5 min at RT (20°C–23°C).
71. Aspirate off the washing media and resuspend the pellet in 4 mL warm wash media and spin down the cells again at 150 × *g* for 5 min at RT (20°C–23°C).

***Note:*** For all washing steps, the cell pellet should be broken up using only the stream of media from a p1000 pipette set to 1000 μL, if at all possible, as the loss of cells tends to be substantial if aspirations are required, as they will stick to the plastic of the pipette tip.

72. Repeat the washing step once more.
73. During the wash steps, prepare 6-well culture plates (non-tissue culture-treated for the CC and SVZ-derived NSCs, and tissue adherence culture-treated plate for the SGZs-derived NSCs from P1 and onwards) for seeding by adding 3 mL of culturing media to the new wells and place the plate in a humidified incubator (5% CO_2_, 37°C).
74. Following the second wash, remove the wash media and resuspend the pellet in 1 mL culturing media.

**CRITICAL:** Carry out the next steps quickly to ensure cell viability does not decrease.

75. Take 10 μL of resuspended cell solution and add this to 10 μL of 0.4% Trypan Blue in a 500 μL Eppendorf tube. Mix gently but thoroughly (about 10 aspirations) and apply 10 μL to both sides of a cell counting chamber slide.
76. Count the cells using an automatic cell counter (or other method of choice) and record the numbers from both sides of the chip.

**CRITICAL:** Double the number of wells on each passage to give the cells space to expand. Seeding density should not exceed 5 × 10^6^ cells per well.

77. Split the cell solution evenly between the new wells and return the plate to the humidified incubator (5% CO_2_, 37°C).

***Note:*** For best results make sure that cells are evenly distributed throughout the wells. One option here is to gently swirl the plate, switching between clockwise and counterclockwise directions a number of times.

### Main procedure: NSC characterization

**Timing: 3 days**

This step presents a method for labeling and characterizing the cells resulting from the successful application of the protocol through EdU incorporation into the DNA of actively proliferating cells and immunocytochemical labeling for NSC and neural differentiation cell markers. We have chosen an array that tests for broadly recognized NSC characteristics (self-renewal and multipotency that should be applicable to NSCs from all three neurogenic niches. As the lateral SVZ NSCs primarily differentiate into neurons and astrocytes (Mizrak et al., 2019) and SGZ-derived NSCs practically do not differentiate into oligodendrocytes *in vivo* (Wachs et al., 2003), oligodendrogenesis was not tested for as a measure for multipotency beyond the expansion stage *in vitro*.

78. Dilute EdU in relevant cell media to a 1:500 dilution and replace 50% of cell media with EdU media solution, resulting in a final EdU solution of 1:1000 per well (10 μM/mL). Leave cells in EdU solution for the desired duration, 12–24 h recommended.

Fixation (30 min).

79. Remove media and submerge cells in 3% glyoxal solution (Richter et al., 2018) for 15 min (300 μL/well in 24 well plates, adjust volume to vessel used to ensure complete coverslip submersion).
80. Remove Glyoxal solution and wash twice with 1× PBS for 5 min each, increasing volume of PBS by 100 μL for each wash.

***Note:*** If more than 1 plate is being processed, start timers once the first plate has been completed, not the last for more accurate times (Do this for all steps).

81. The immunostaining process can be started immediately after fixation, or the plates can be stored at 4°C for up to 3 weeks in 1× PBS, however the quality of the cells will start deteriorating after 1 week.

Immunostaining (2 days).

Day 1: Primary antibodies (20–24 h).

82. Remove PBS from wash and replace with 0.5% Triton-X 100 in 1× PBS for 5 min.
83. Remove Triton-X and wash twice for 5 min with 1× PBS, increasing volume of PBS for each wash.
84. Replace PBS with 5% Goat Serum diluted in 1× PBS (blocking solution) and set aside for 1 h (300 μL/well is enough for 24 well plates). Prepare primary antibody solution in 5% goat serum at this time and store in fridge until needed.
85. Remove goat serum blocking solution and submerge cells with primary antibody solution. Transfer plates into the cold room and place onto shaker table and leave overnight (18–22 h, at 4°C).

***Note:*** Always leave 1 well without primary antibodies for a primary antibody exclusion control.

Day 2: Secondary antibodies and sample preservation (6 h).

86. Prepare secondary antibodies (1:500 standard solution) in 5% goat serum in 1× PBS.
87. Centrifuge secondary antibody solution at 2,000 × *g* for approx. 15 min, perform washing steps during this step.
88. Remove primary antibody solution and wash with 1× PBS 3× times for 5 min each, increasing volume of PBS for each wash.

***Note:*** Always start each wash/step from here with the primary exclusion control and change tips between each solution and step (the more the better to avoid cross- contamination of antibodies. It is recommended to use a separate PBS and waste vial for the primary exclusion.

89. Remove PBS and submerge cells in the secondary solution.
  a. Place on a shaker table set to low speed and leave for 3 h, protected from light.
  b. Take out Prolong Gold Antifade mounting media at this time and leave to warm at room temperature.
90. Prepare Hoechst solution (1:2000) in 1× PBS and protect from light during the last 30 min of the secondary incubation time.
91. Remove secondary solution and submerge cells in the Hoechst solution for 30 min (300 μL/well).
92. Remove Hoechst solution and wash 3× times with 1× PBS, increasing the volume of PBS for each wash.
93. Wash 1× additional time with MQ water. Remove MQ water and replace with new MQ water (700 μL/well for 24 well plate).
94. Prepare cover slide, mark with date, sample type and secondary antibody wavelength.
95. Add a small drop of mounting media onto the cover slide. Remove coverslip from the well with fine forceps and dry by gently tapping one edge onto a sheet of tissue paper. Mount the coverslip by touching the mounting media with one side of the coverslip, then slowly lowering the other side, ensuring that no air bubbles are trapped under it.

***Note:*** Use Dumont 5/90 curved forceps to easier grab hold of the coverslips. Hold the plate at approximately 75°–80° so that the coverslip can rest in a standing position for ease of grabbing. Slowly lower the coverslip onto the mounting media, be careful not to get mounting media on top of the coverslip as this will introduce a lot of background and potentially ruin the sample. To ensure the coverslip is properly mounted (level), check it with a fluorescent microscope at low magnification (narrow/low numerical aperture recommended) for DAPI/Hoechst with the coverslip facing upwards, and adjust with forceps.

96. Leave the coverslips to dry for 20–30 min, then gently seal the edge with clear nail varnish topcoat, ensuring minimal varnish on top of the coverslip.

***Note:*** Do not use nail polish and colored/matte nail polish as this will introduce a lot of background fluorescence.

97. Let nail varnish dry for 15–20 min. Ensure that it is completely dry before handling further.

***Note:*** To check if mounting media/sealant is completely dry, gently touch with a filter paper where it does not cover the coverslip, and when no mark/indentation is left behind the sample is ready for imaging. Sealing with nail varnish is not required but will significantly extend the lifetime of the sample for future imaging, when the sample is stored at 4°C protected from light.

### Main procedure: Additional methods

Freeze-Thaw of NSCs.

Freezing and thawing of the NSCs follow established protocols and can be found elsewhere.

Scanning electron microscopy (SEM).

Protocols for preparing cells for imaging with SEM can be found elsewhere.

Differentiation of CC, SVZ and SGZ-derived NSCs (18+ days).

98. Seed cells onto PLO-Laminin (Table 7) (swap to table 7) coated 13 mm coverslips (seeding density: Table 6) and allowed to expand for 3 days with supplemented expansion media Tables 9 and (10).

**Table 9:** Differentiation base media

Reagent	Final concentration	Amount
Neurobasal 1× Base medium	N/A	47.495 mL
B27 Plus	1×	1 mL
N2	1×	0.5 mL
L-Glutamine	1×	0.5 mL
Heparin	2.5 μg/mL	0.5 μL
**Total**		**50 mL**

Can be stored at 4°C for up to 1 week.

**Table 10:** Differentiation (initial expansion) supplements

Reagent (stock conc)	Final concentration	Amount
EGF (10 μg/mL)	40 ng/mL	12 μL/well
bFGF (10 μg/mL)	40 ng/mL	12 μL/well
CHIR 99021 (10 mM)	10 μM	3 μL/well

Use immediately, do not store for any duration except warming as this will impact growth factor stability.
99. Replace expansion media with differentiation base media (Table 9) supplemented with BDNF and FBS (Table 11).

**Table 11:** Differentiation supplements

Reagent (stock conc)	Final concentration	Amount
BDNF (10 μg/mL)	10 ng/mL	N/A
FBS	1:1000	N/A

Use immediately, do not store for any duration except warming as this may impact neurotrophic factor stability.
100. Keep cells on differentiation media for minimum 15 days, changing media every 3 days.
101. Proceed from step 79.

**CRITICAL:** This is a generalized differentiation protocol, and we recommend using differentiation protocols optimized for each neurogenic niche and desired cell type outcome, as described in troubleshooting 5.

## Expected outcomes

Considering the relevance of neural stem cells in studies of neuroplasticity and regeneration, the ability to extract, maintain, and potentially differentiate these cells from all three canonical neurogenic niches can enable a range of relevant lines of research, including CNS injury and disease modeling.

### Central canal (CC)

After 20 days of expansion, clean monolayer cultures of approximately 2–4 × 10^6^ actively proliferating (EdU and Nestin-positive, GFAP, SOX2, DIx2 and A2B5 negative, Figures 5D, 6B–6E, 7A, and 7B) NSCs can be expected with > 95% of them undifferentiated (GFAP, Beta-3-Tubulin and Map2 negative, Figures 8A and 8B) within the culture when dissociated using accutase. Dissociating these NSCs using trypsin will result in a negative rate of proliferation ending in non-viable cultures by P2 (Figure 5A). These NSCs do not spontaneously differentiate towards a neural fate unless actively induced and can robustly survive and proliferate on the greatest variety of substrates of all the three neurogenic niche-derived NSCs (Figures 5C, 6E, 7A, and 7B). Adding CHIR 99021 to the CC-derived cultures will induce differentiation (Figure 9), even when cultured in expansion media, and should only be added when starting differentiation.

### Subventricular zone (SVZ)

After 20 days of expansion, a clean culture of either approximately 4–5 × 10^6^ (accutase; EdU, Nestin, SOX2 and few GFAP-positive, DIx2 and A2B5 negative, Figures 5A, 7C, and 7D) or 1.4–1.6 × 10^7^ (trypsin; EdU, Nestin, SOX2 and GFAP-positive, DIx2 and A2B5 negative, Figures 10A and 10B) actively proliferating NSCs developing as settled colonies from P1 (Figure 6G–6J (accutase)). During expansion these cells are highly sensitive to the surface characteristics of the culture vessel used and will only expand and retain proper NSC characteristics when cultured on a smooth surface, such as non-tissue culture treated plates. On uneven and more coarse surfaces, such as tissue culture treated plates or uncoated glass coverslips, the NSCs will start to spontaneously differentiate and die, resulting in complete culture termination within short time (Figure 5C). When cultured on PLO-Laminin coated glass coverslips survival resembles that of smooth surface culturing. A small subpopulation of the NSCs cultured on coated glass coverslips may spontaneously differentiate towards a neural fate (10%–20% GFAP, Beta-3-Tubulin and Map2 positive, Figure 7C). Trypsin-dissociated NSCs results in cultures similar to those derived from accutase-dissociated tissue, however, they display a higher rate of proliferation (Figure 5A) and are much more likely to adopt a glial, rather than neuronal fate. Observed NSC GFAP positive glia (i) accutase: 5, SD = 3.8. (ii) trypsin: 133, SD = 64.1. p = 0.004 two-tailed. Average number of cells (Hoechst positive) (i) accutase: 4585, SD = 3022. (ii) trypsin: 4950, SD = 2083. p = 0.81 (two-tailed). Observed Differentiated GFAP positive glia (i) accutase: 9, SD = 5.9. (ii) trypsin: 416, SD = 149.9. p = 0.001 (two-tailed). n = 6 observations per condition, observed across 4 accutase-treated and 2 trypsin-treated harvests. See quantification and statistical analysis section for details. Illustrated in Figures 10C and 10D). Neither of the accutase or trypsin-dissociated SVZ-derived NSC cultures contain Type-C transit amplifying cells (Dix2 negative, Figures 7D and 10B), which may be explained by the presence of EGF within the growth media (Doetsch et al., 2002).

### Subgranular zone (SGZ)

After 20 days of expansion, a clean culture of approximately either 1 × 10^7^ (accutase) or 1.2–1.4 × 10^7^ (trypsin) actively proliferating (EdU, Nestin, GFAP-positive, SOX2, DIx2 and A2B5 negative, Figures 5A, 7E, and 7F) NSCs can be expected with > 85% undifferentiated (GFAP-positive, A2B5, Beta-3-Tubulin and Map2 negative) NSCs within the culture, developing as monolayer colonies (Figures 6L–6O and 8E). During expansion these cells are highly sensitive to the surface characteristics of the culture vessel used and will only expand and retain proper NSC characteristics when cultured on a coarse, uncoated surface post P1 passage, such as tissue culture treated plates. On smooth surfaces, such as on non-tissue culture treated plates, a small subpopulation of the NSCs will start to spontaneously differentiate before dying, with extensive cell death for the rest (Figure 5D). When cultured on PLO-Laminin coated glass coverslips, a small subpopulation of the NSCs will spontaneously differentiate into large GFAP-positive radial glia-like cells, seemingly providing a scaffolding for the remaining Nestin-positive NSCs (Figures 6O and 8E). However, proliferation rate and survival will be drastically lowered in comparison to proper expansion conditions (Figures 6O vs 7E). After a short period of differentiation (< 7 days) an increase in cell proliferation may occur.

## Quantification and statistical analysis

Cell proliferation and survival: Cell numbers and survival percentage were counted automatically using a Countess™ II Automated Cell Counter (Invitrogen, AMQAX1000) and averaged.

For SVZ accutase and trypsin GFAP quantification: Cells were counted using FIJI’s (ImageJ) automated cell counting function based on ICC images (Hoechst and GFAP, 0.5 cm^2^ field of view per image, 6 images per condition, acquired with EVOS M5000, Thermo Fisher) of the cell cultures. Data was then tested for equal variance before t-tests (Student’s & Welch’s, Microsoft Excel, 2016) were performed to determine any difference between number of GFAP-expressing cells in the accutase and trypsin-dissociated cultures.

## Limitations

### Age of the animals

The protocol was optimized for extracting NSCs from 10-week-old Female Sprague Dawley rats, other strains and ages might yield different survival and expansion rates. With older animals it is likely that NSC yields will be reduced, while younger animals will likely produce greater yields more quickly.

### Cell sensitivity to culture vessel surface

NSCs from all three niches displayed a high degree of sensitivity to the material and surface treatment of culture vessel on which they were expanded. We have described which vessels and surface treatments produced the best and most reliable results. If these are not available, it may be necessary to adjust the assay as variability in the surface quality of the culture vessels available from different manufacturers cannot be excluded.

### Seeding density for expansion

Several initial seeding densities were assessed in optimizing this protocol; 5 × 10^5^ live cells per well of a 6-well plate (5.2 × 10^4^ cells/cm^2^) resulted in the most consistent and reliably high rate of expansion for the age and strain used. This may not necessarily be the ideal seeding density for different animal ages and strains. Adjustments can be considered if the rate of proliferation is too high or not sufficient when changing these parameters.

### NSC characterization

As there is currently no consensus in regards to NSC identity (Bazan et al., 2004; Chojnacki et al., 2009; Ghosh 2019; Massirer et al., 2011), or universally recognized NSC-specific markers (Bazan et al., 2004) in addition to multiple distinct cell types that exhibit NSC characteristics in the literature (Mizrak et al., 2019), NSC characterization should be based upon the main hallmarks of NSCs: active proliferation (EdU/BrdU/Ki67), at least one recognized NSC marker (Nestin/SOX2 etc.), and neural lineage multipotency (GFAP/MBP1/Map2/NFH/B3T). Controversy also exist regarding adult NSC multipotency, and this should be taken into account when working with these cells (Bond et al., 2015).

When adapting this protocol to other strains/species (mice) it is important to take species differences, even for NSCs, into consideration when characterizing the cells. Of particular importance is the fact that 0%–10% of rat-derived NSCs express GFAP (Figure 7), while 60%–80% of mouse-derived NSCs express GFAP (Steffenhagen et al., 2011).

### Imaging

As the optimal growth substrate for the NSCs (particularly SVZ and SGZ) is difficult to replicate on coverslips, it is recommended to perform immunolabeling for characterization within the original culture wells. For imaging it is then required to have specialized microscope objectives capable of imaging through the bottom of a culture well plate, and this may limit maximum achievable magnification.

## Troubleshooting

### Problem 1

Low yield of live/viable NSCs following dissociation (steps 39–53).

### Potential solution

Practice of difficult steps (e.g., dissection technique and speed on cadavers) can help decrease the amount of time spent between animal euthanasia and completing the dissociation, which contributes to higher cell survival. While the protocol can be carried out by one person, dividing tissue extraction and dissociation between two people can help speed up the process. All preparation steps for the entire process from starting the dissections to finishing the dissociation should be completed before starting euthanasia of the animal. Generally, it is a good idea to identify the steps that are the most complicated and first try to practice these to optimize the workflow. Additionally, the following factors were observed to have an impact on cell viability: (i) The tissue needs to be kept cold during the entire dissection, any warming especially after it has been removed and before the dissociating agent is added will have a deleterious effect. (ii) Insufficient cutting of the tissue can lead to either too large chunks or not fully separated pieces of tissue. These will make the mechanical dissociation more difficult and will substantially reduce yield and viability. (iii) White matter that is accidentally included in the dissected tissue can have a similar negative effect. (iv) Incubation time with the dissociating agent should not exceed 15 min with accutase or 5 min with trypsin. (v) It may be necessary to switch hands or operate the pipette with a different finger to avoid fatigue and keep the mechanical dissociation (step 43) to a short enough time for optimal survival. (vi) If problem persists with cultures dissociated using trypsin, use of trypsin-inhibitors during neutralization steps (44–46) is recommended.

### Problem 2

Excess cell/tissue debris in culture following dissection (steps 54–61).

### Potential solution

Ensure that as much of the media as possible is aspirated off during each P0 media change. Triturate the neurospheres 4–6 times using the serological pipette to clean them and to break up debris. Aspirate as much of the supernatant as possible. The pellet formed by the centrifuged neurospheres will keep the neurospheres in place provided that no excessive force is used during aspiration. Make sure to resuspend with new media quickly to avoid unnecessary cell loss. Repeat when resuspending the neurospheres. 90%–95% of debris should be gone following the P1 passage. Once the cells have attached following the P1 passage they can be gently rinsed with warm media without detaching for further cleaning. This should only be done if absolutely necessary.

### Problem 3

Slow proliferation rate of NSCs (steps 54–77).

### Potential solution

As growth factors are critical for sustained proliferation, expansion media should always be freshly supplemented before a media change or passage. Growth factor aliquots should not be used if they have been stored at 4°C for over 7 days. Frozen stocks should not be used if they have surpassed the shelf life noted by the manufacturer. Growth factors are generally more stable when frozen in solution also containing 0.1% filtered Bovine Serum Albumin (BSA). In addition, consider testing different cell seeding densities on different culture vessel topographies to find optimal combinations for cell growth.

### Problem 4

Poor cell adhesion/monolayer formation post P1 passage (steps 54–77).

### Potential solution

Make sure the cell culture vessels are kept stably and without disruption for 4 days following passaging to allow the cells to settle on the bottom of the wells. Take particular care not to damage/scratch the surface of the well as this will have a significant impact on survival vs proliferation vs auto-differentiation. An increase of growth factor (EGF & bFGF) concentration can also help induce cell settling and proper monolayer formation. This should be optimized through a concentration titration test, and it is recommended to not increase the concentration of growth factors by more than 10 ng/mL for each step.

### Problem 5

Poor/undesired yield of differentiated cell types (steps 98–101).

### Potential solution

Optimize differentiation protocol to the desired cell type to be differentiated as the NSCs derived from each of the neurogenic niches can be differentiated into different neural/neuronal cell types and relative proportions within each culture by adding different factors to the media. Age of the NSCs, in addition to whether they have been frozen, can also impact their sensitivity to compounds such as FBS and should be tested whenever starting differentiation.

### Problem 6

Extensive cell death/non-viable SGZ-derived NSC cultures (steps 54–77).

### Potential solution

SGZ-derived NSCs have shown positive response to the addition of vascular endothelial growth factor (VEGF) to the cell media in other studies. If extensive cell death (can accompany the rapid proliferation or culturing on glass coverslips) is a problem, then tests can be done with VEGF as a potential addition/alternative to CHIR 99021 (note: This has not been tested as part of this protocol) (During and Cao, 2006; Han et al., 2015; Schänzer et al., 2004; Segi-Nishida et al., 2008). The use of VEGF should be limited as it can cause the NSCs to adopt tumorigenic properties during expansion (Kaus et al., 2010).

## Resource availability

### Lead contact

Further information and requests for resources and reagents should be directed to and will be fulfilled by the lead contact; Professor Ioanna Sandvig (ioanna.sandvig@ntnu.no).

### Materials availability

This study did not generate any new unique reagents.

## Data Availability

No unique datasets were generated for this protocol. No code was generated from this study.
